# *DNAH9* variants in children with post-infectious bronchiolitis/bronchitis obliterans

**DOI:** 10.1186/s13023-025-03616-4

**Published:** 2025-03-10

**Authors:** Yuhong Guan, Xiaoyan Zhang, Xiaolei Tang, Haiming Yang, Shunying Zhao

**Affiliations:** Department II of Respiratory Medicine, National Clinical Research Center for Respiratory Disease, Beijing Children’s Hospital, Capital Medical University, National Center for Children’s Health, Beijing, China

**Keywords:** DNAH9 variants, Post-infectious bronchiolitis/Bronchitis obliterans, Children, *Mycoplasma pneumoniae*, Adenovirus

## Abstract

Post-infectious bronchiolitis/bronchitis obliterans (PIBO) is a chronic irreversible obstructive lung disease that results in obstruction and/or obliteration of small airways. Previous reports have indicated that PCD-related gene mutations contribute to PIBO incidence. However, the relationship between *DNAH9* variants and PIBO remains unclear. This study aimed to evaluate the association between *DNAH9* mutations and the incidence of PIBO. In our cohort, 126 PIBO patients conducted Whole Exome Sequence (WES) test and twelve variants of DNAH9 gene were identified. Detailed clinical information, high-resolution computerized tomography and/or electronic bronchoscopy findings of the six pediatric children carried DNAH9 variants were systematically collected, meticulously reviewed, and rigorously analyzed. Clinical evaluation revealed three patients with bronchiolitis obliterans, two patients with bronchitis obliterans and one with both conditions. All patients had at least one previous bout of pneumonia, which in three cases was linked to *Mycoplasma pneumoniae*, in two cases to adenovirus infection, and in one case to co-infection with both pathogens. Genetic analysis of all cases identified six compound heterozygous *DNAH9* mutations encompassing twelve variants: c.12,925 C > T (p.Arg4309*), c.5152-10G > T (-), c.4604 A > G (p.Gln1535Arg), c.12844-14T > C (-), c.4816T > C (p.Phe1606Leu), c.8831G > A (p.Arg2944Gln), c.9479 C > T (p.Ala3160Val), c.7415G > A (p.Arg2472Gln), c.5692G > T (p.Glu1898*), c.11,572 C > T (p.Arg3858Trp), c.11,176 C > T (p.Arg3726Trp), c.1010 C > T (p.Pro337Leu). These variants included two nonsense mutations, two mutations near splice sites, and eight missense mutations. All variants exhibited negligible or low minor allele frequencies based on the gnomAD database and were predicted to be variants of uncertain significance (VUS) or deleterious based on comprehensive bioinformatics analysis. Our findings suggest that *DNAH9* compound complex variants may contribute to development of PIBO following severe *M. pneumoniae* and/or adenoviral infectious pneumonia in pediatric patients.

## Introduction

Primary ciliary dyskinesia (PCD) is a rare genetic disorder characterized by considerable genetic and clinical diversity. Common clinical manifestations include chronic wet cough, recurrent wheezing, perennial nasal congestion, and laterality defects, with bronchiectasis affecting over 80% of PCD patients [1]. To date, more than 50 PCD-related genes have been identified [2]. Among these, *DNAH9*, which encodes a heavy chain of axonemal dynein, plays a pivotal role in ciliary motility by harnessing energy derived from ATPase-induced ATP hydrolysis to enable the generation of a sliding force by motile cilia. Notably, *DNAH9* mutations have been associated with PCD in individuals presenting with situs abnormalities, mild respiratory manifestations, and severe asthenospermia [3–5]. Additionally, *DNAH9* polymorphisms have been linked to asthma and bronchial hyperresponsiveness, particularly in response to early-life exposure to tobacco smoke [6].

Post-infectious bronchiolitis/bronchitis obliterans (PIBO) is a chronic obstructive pulmonary disease often arising after childhood bouts of severe lower respiratory tract infections, with PIBO susceptibility influenced by complex genetic and environmental factors that remain poorly understood [7]. Various hypotheses, including genetic predisposition (‘risk alleles’) and immune dysregulation, have been proposed as potential contributors to PIBO development [8, 9]. For example, Giubergia et al. reported an increased frequency of polymorphisms in the *mannose-binding lectin 2* (*MBL2*) gene in PIBO patients as compared to healthy controls, underscoring the significant role of genetic factors in PIBO pathogenesis [10]. Similarly, variants of other PCD-related genes, such as *CCDC39* and *DNAH1*, have been implicated in PIBO development [11, 12], with rapid advancements in sequencing techniques expected to lead to accelerated identification of novel disease-causing gene variants. This study explores the possible impact of *DNAH9* variants on PIBO progression in children.

## Materials and methods

### Subjects

A hospital-based retrospective analysis was conducted on data for 126 children with PIBO and 118 were performed WES test at Beijing Children’s Hospital from during December 2017 to February 2023. Twelve variants of DNAH9 were identified in six patients. Clinical information, including detailed medical histories and findings of physical and accessory examinations, was collected by experienced pediatricians. The study protocol was reviewed and approved by the Ethics Committee of Beijing Children’s Hospital, China (Approval no. (2024)-Y-090-D). Informed consent was obtained from the parents of all study participants.

The diagnosis of post-infectious bronchiolitis obliterans was established based on the following diagnostic criteria: (1) history of an acute and severe lower respiratory tract infection; (2) repeated or persistent cough, wheezing, or shortness of breath over the 6 weeks after acute infection with decreased exercise tolerance; (3) mosaic perfusion signs, air trapping, bronchial wall thickening, bronchiectasis, or atelectasis on chest HRCT; (4) obstructive ventilatory dysfunction on pulmonary function tests; (5) exclusion of other chronic lung diseases occurring prior to BO onset, such as asthma and bronchopulmonary dysplasia [13, 14].

In this study, a history of *Mycoplasma pneumoniae* (MP) infection was confirmed if both of the following criteria were met: (1) serum anti-MP IgM titer ≥ 1:320 and/or ≥ 4-fold increase in anti-MP IgM titre between acute and recovery stages; (2) positive results of MP polymerase chain reaction (PCR) testing of pharyngeal swabs. Patients were diagnosed with adenovirus (ADV) pneumonia based on positive respiratory multiplex PCR test results obtained for adenovirus.

### Genetic analysis

**Fig. 1:**
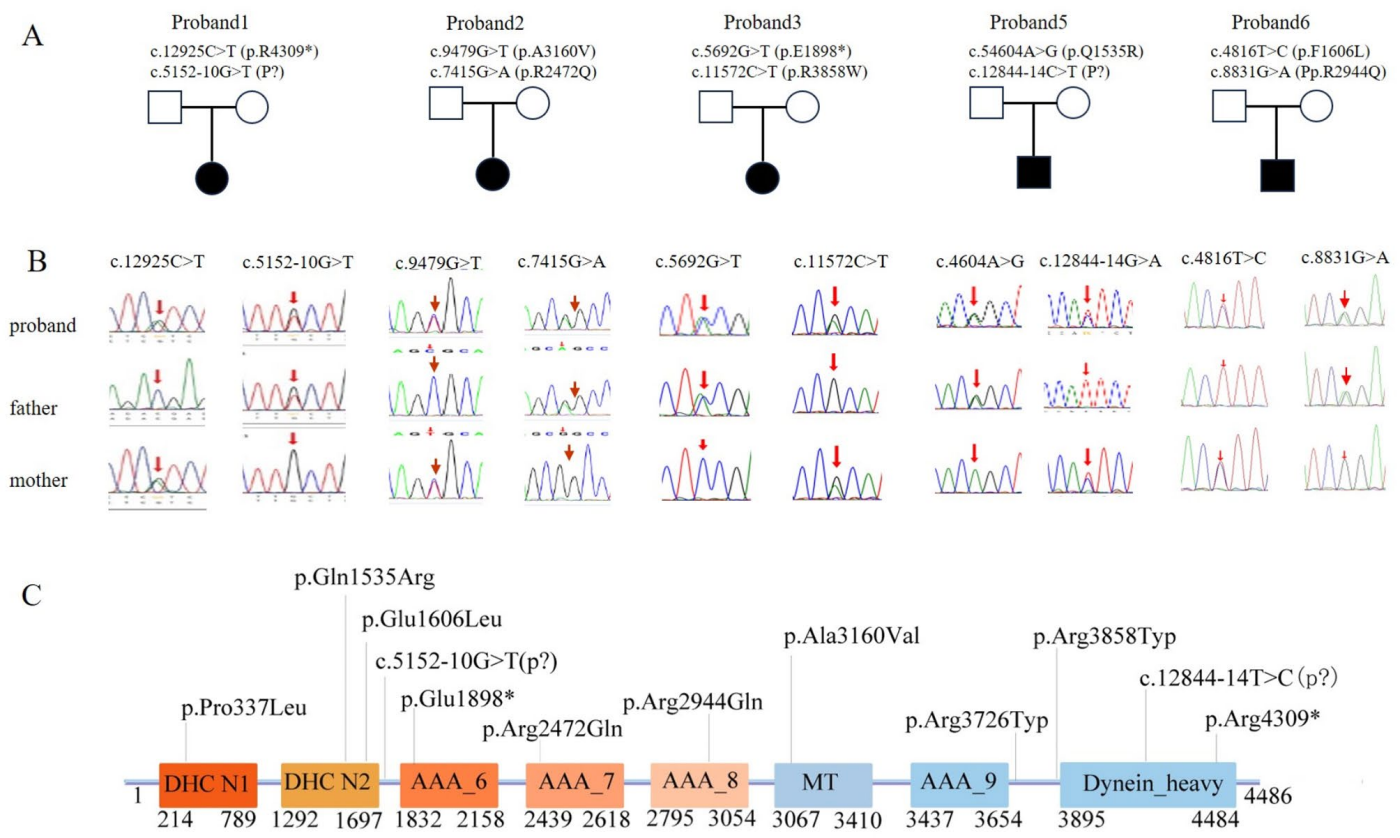
Sanger confirmation and mapping of rare damaging mutations in the DNAH9 genes in five subjects. (**A**) Pedigrees of families 1–3,5,6 indicating the affected individuals and the segregation of *DNAH9* recessive variants. (**B**) Sanger sequencing on DNAH9 variants in the patients and their unaffected parents of patient 1–3,5,6. (**C**) Predicted protein model of DNAH9 with the distinct domains and indication of DNA fragment distribution for protein-protein interaction experiments

We performed WES on affected individuals followed by validation of segregation of candidate variants through analysis of parental variant profiles using Sanger sequencing (Fig. 1b and c). Variant filtering was performed based on a minor allele frequency (MAF) cutoff of < 0.01 in the gnomAD database as previously reported [11]. After genomic DNA samples were prepared, exonic DNA sequences were enriched using the NimbleGen 2.0 probe sequence capture array (Roche) then sequencing was performed using the Illumina HiSeq2500 platform (Illumina). Raw sequence data underwent quality assessment using the manufacturer’s recommended base-calling method then low-quality sequence reads (quality scores < 20) were discarded. The remaining sequence reads were aligned to the National Center for Biotechnology Information human reference genome (hg19) using Burrows-Wheeler alignment.

Variant calling based on single-nucleotide polymorphisms and insertions and deletions was performed using SAMtools and Pindel, respectively. Candidate missense mutations were evaluated for pathogenicity using SIFT (http://sift.bii.a-star.edu.sg/), PolyPhen-2 (http://genetics.bwh.harvard.edu/pph2/), and Mutation Taster (http://www.mutationtaster.org/). Splice-site changes were assessed using MaxEntScan (http://hollywood.mit.edu/burgelab/maxent/Xmaxentscan_scoreseq.html) and dbscSNV (http://www.liulab.science/dbscsnv.html) software packages. Variant evaluation followed American College of Medical Genetics and Genomics (ACMG) guidelines. Candidate causal variants of *DNAH9* (GenBank: NM_001372.3) were confirmed using Sanger sequencing before parental carrier screening was performed.

## Results

### Clinical features of PIBO patients with *DNAH9* variants

In this study, we enrolled six children diagnosed with bronchiolitis/bronchitis obliterans, along with the parents of five of them, resulting in the inclusion of five unrelated family trios and one sporadic case without parental samples for segregation analysis (Table 1; Fig. 1). Detailed clinical information and genetic results of recruited PIBO patients and parents were systematically reviewed; all recruited parents were unaffected and healthy (Fig. 1).

**Table 1:** Clinical features of the individuals with DNAH9 variants

	P1	P2	P3	P4	P5	P6
Male/Female	F	F	F	F	M	M
Age at present(y)	5	9.8	3.1	14.3	4.4	1
History	-	-	-	-	-	-
MP	+	-	-	/	+	-
Pulmonary						
Bronchiectasis	N	Y	Y	Y	Y	N
Bronchiolitis/bronchitis obliterans	Y	Y	Y	Y	Y	Y
Atelectasis	Y	N	N	N	Y	N
PFT	Normal	Obstructive	Obstructive	MIX	NA	Obstructive
FEV1/R_5_ (%pred)	84.7	77.1	162.4	15.3	-	-
FVC/R_20_ (%pred)	85.5	82.3	73.5	50.9	-	-
FEF_25 − 75_ (%pred)/R_5 − 20_	67.2	49.9	1.31	5.0	-	-
nNO (nl/min)	136.4	98.6	108.8	331.8	/	48.9
Athma	N	Y	N	N	N	N
NRD	N	N	N	N	N	N
SI	N	N	N	N	N	N
Sinusitis	Y	N	N	N	Y	N
Otitis media	N	N	N	N	N	N
Hearing loss	N	N	N	N	N	N

MP: *Mycoplasma pneumoniae;* PFT: pulmonary function test; nNO: nasal nitric oxide; NRD: Neonatal respiratory distress; SI: situs inversus; NA: not available

Phenotypic characteristics of patients are summarized in Table 1. Among the six patients, four were male and two were female, with PIBO ages of onset ranging from 11 months to 13 years. The observed average age of presentation was 6.1 years.

In our cohort, five patients presented with an acute course of cough and fever (patients P1, P2, P3, P5, P6), with three of them also experiencing wheezing. The remaining patient presented with cough, dyspnea, and decreased exercise tolerance (P4). Patient P1 had a history of wheezing and skin allergy. Notably, none of the six patients exhibited signs of situs inversus (SI) or heterotaxy syndrome, while sinusitis was observed in three patients. Results of allergen tests and immunological assays were within normal ranges for all six patients. Three of the six patients were confirmed to be infected with *M. pneumoniae*, two with adenovirus, and one with both M. pneumoniae and adenovirus.

**Fig. 2:**
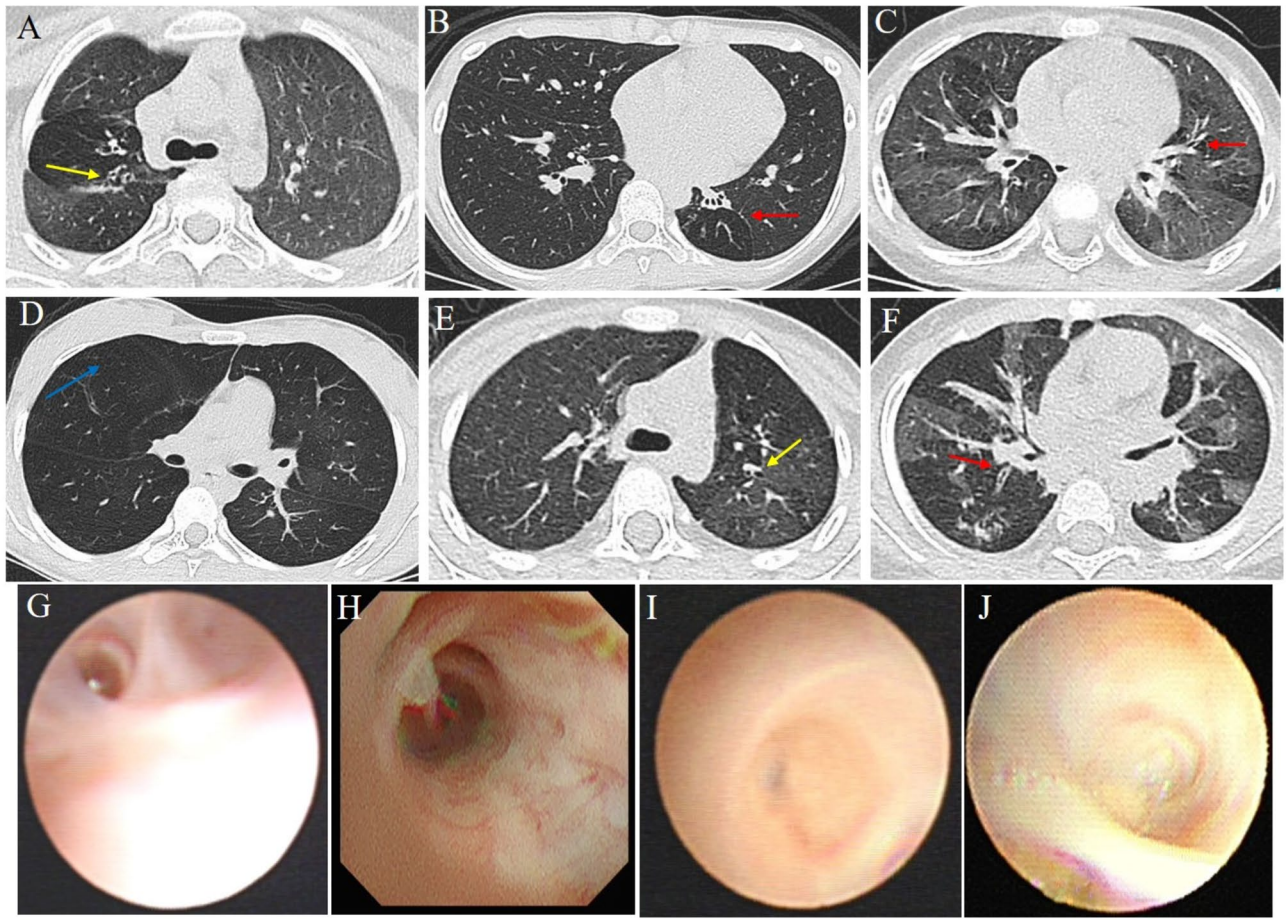
Thorax CT imaging and of the six study subjects (**A**-**F**). Upper and lower rows indicate patient (1–6) images, respectively. Red arrows indicate mosaic attenuation patterns and Bronchiectasis. Yellow arrow indicates a large bronchiectasis and bronchial wall thickening. Blue arrow indicate emphysema. Bronchoscopy revealed presence of subsegmental bronchitis obliterans in four patients (**G**-**J**)

Nasal nitric oxide (nNO) levels were within the normal range for four patients, whereas one patient exhibited a relatively lower level (48.9 nl/min) than the typical cut-off value for PCD used in prior studies (77 nL/min). The nNO level of the remaining patient was not determined. HRCT showed mosaic perfusion, air-trapping, bronchial wall thickening in the lungs, confirming the diagnosis of bronchiolitis obliterans in three patients (Fig. 2). Additionally, bronchoscopy findings revealed the presence of subsegmental bronchitis obliterans and bronchiectasis in four patients, while pulmonary function tests revealed obstructive ventilation dysfunction in four patients (Fig. 2).

### Genetic analysis

WES analysis was conducted to identify potential PIBO-related variants, resulting in detection of twelve compounds heterozygous *DNAH9* variants in six unrelated patients. These variants included the following: c.12,925 C > T (p.Arg4309*), c.5152-10G > T (-), c.4604 A > G (p.Gln1535Arg), c.12844-14T > C (-), c.4816T > C (p.Phe1606Leu), c.8831G > A (p.Arg2944Gln), c.9479 C > T (p.Ala3160Val), c.7415G > A (p.Arg2472Gln), c.5692G > T (p.Glu1898*), c.11,572 C > T (p.Arg3858Trp), c.11,176 C > T (p.Arg3726Trp), c.1010 C > T (p.Pro337Leu). Among these variants, two were nonsense mutations, two were near splice acceptor sites, and eight were missense variants. Eight variants were classified as likely pathogenic variants, while four were deemed of uncertain pathogenic significance based on ACMG guidelines (Table 2).

**Table 2:** The pathogenicity of DNAH9 variants in PIBO patients

	cDNA change (Protein change)	dbSNP	Source	GnomAD (East Asian)	Poly-phen2 score	Mutation- Taster score	SIFT Score	ACMG	Ref
P1	c.12,925 C > T(p.R4309*)	NA	M	0.00007	-	-	-	P	Current
	c.5152-10G > T(P? )	NA	P	None	-	-	-	VUS	Current
P2	c.9479 C > T(p.A3160V)	rs770572516	M	None	1.000 Pro-D	0.999 D	-3.765 D	VUS	Current
	c.7415G > A(p.R2472Q)	rs188772907	P	0.00089	0.034 B	0.726D	-2.079 N	VUS	Current
P3	c.5692G > T(p.E1898*)	-	P	None	-	-	-	P	Current
	c.11,572 C > T(p.R3858W)	rs779372233	M	0.00002	1.000 Pro-D	0.999 D	-7.480 D	VUS	Current
P4	c.11,176 C > T(p.R3726W)	rs3760436	-	0.0063	1.000 Pro-D	0.999 D	-7.517 D	VUS	35,050,399
	c.1010 C > T(p.P337L)	Rs3744574	-	0.0056	0.026 B	0.999 D	-5.815 D	VUS	Current
P5	c.4604 A > G(p.Q1535R)	rs147183329	P	0.0034	1.000 Pro-D	0.999 D	-3.664 D	VUS	Current
	c.12844-14T > C(P? )	NA	M	0.00072	-	-	-	VUS	Current
P6	c.4816T > C(p.F1606L)	rs765169895	M	0.00033	0.884 Pro-D	0.999 D	-5.474 D	VUS	Current
	c.8831G > A(p.R2944Q)	rs550801585	P	0.0010	0.909 Pro-D	0.625 D	-2.851 D	VUS	Current

D: Deleterious; N: Neutral; B: Benign

Pathogenicity analysis of *DNAH9* variants conducted using SIFT and Mutation Taster bioinformatics tools revealed that all eight *DNAH9* missense variants were deleterious with regard to disease-causing potential, while PolyPhen-2 analysis of these variants predicted that six of them were probably deleterious. Notably, c.1010 C > T (p.Pro337Leu) and c.7415G > A (p.Arg2472Gln) missense variants affected evolutionarily highly conserved amino acids within the DNAH9 dynein heavy chain 1 (DHC1) and ATPase domains, respectively. Similarly, the c.8831G > A (p.Arg2944Gln) and c.9479 C > T (p.Ala3160Val) missense variants affected amino acids within the AAA + ATPase domain, while c.12,925 C > T (p.Arg4309*) and c.11,572 C > T (p.Arg3858Trp) variants altered amino acids within the C-terminal domain.

### Parental carrier testing

Parental carrier testing demonstrated that each parent of five of the six patients carried one of the mutations in a trans configuration, indicating an inherited pattern. However, parental samples for patient P6 were unavailable for DNA analysis. Importantly, no mutations related to primary immunodeficiency disease or cystic fibrosis transmembrane conductance regulator (CFTR)-related disorders were identified in these subjects.

## Discussion

In this study, we identified biallelic variants in the *DNAH9* genes of six unrelated Chinese patients with PIBO with the objective of assessing their potential roles as genetic contributor to the incidence of PIBO in children. *DNAH9* encodes an axonemal dynein heavy chain that is critical for ciliary motility. Previous research by Zheng et al. demonstrated that *Dnah9* KD mice exhibited increased mucin secretion, compromised lung function, substantial inflammation and outer dynein arm (ODA) defects [15]. Typically, patients with *DNAH9* mutations present with mild respiratory symptoms, sinusitis, and occasional ciliarelated complex congenital heart disease [3, 4, 16, 17]. However, some *DNAH9* mutations have been associated with chronic wet cough, asthma, bronchial hyperresponsiveness, and even jejunal atresia in rare cases [6, 13, 17, 18]. These findings underscore the considerable phenotypic variability associated with *DNAH9* mutations across individuals.

While sinusitis is a common feature among patients with *DNAH9* mutations, it is noteworthy that four patients in the current study had normal nNO levels despite the presence of ciliary function-disrupting *DNAH9* mutations. This result suggests that residual ciliary activity may be sufficient to maintain normal nNO levels, as consistent with the observation that none of the six patients had experienced recurrent respiratory infections. However, despite their mild respiratory symptoms, all six patients developed PIBO following bouts of severe ADV- and/or MP-infected pneumonia. Interestingly, the *DNAH9* variants identified in these individuals were either absent or occurred at low MAFs (< 0.005) in the gnomAD database, underscoring their potential rarity and clinical significance.

Notably, the c.1010 C > T (p.Pro337Leu) variant identified in patient P6 encodes an amino acid residue found within domain 1 of DNAH9 dynein heavy chain 1 (DHC1) within a region known to interact with full-length CCDC114 and contribute to ciliary dysfunction. Furthermore, the variant c.11,176 C > T (p.Arg3726Trp) has been previously implicated in complex congenital heart disease [16], highlighting its potential pathogenicity. Unfortunately, due to our patients’ poor lung function and young ages, transmission electron microscopy for assessing ciliary structure was not available in this study.

PIBO is a chronic and irreversible obstructive airway disease caused by damage to small airways following a lower respiratory tract infection [19]. Disease pathogenesis has been linked to certain genetic risk factors, including PCD-related genes [20]. Interestingly, inflammatory cytokines such as IL-1α and IL-8 may participate in the development of PIBO and ADV/MP infection-induced pneumonia [21–23]. Moreover, an analysis of immune function-related cytokine profiles of *Dnah9* knock-down (KD) mice demonstrated significantly decreased levels of several cytokines, including GM-CSF, IL-1α, and TNFα [15], suggesting a link between *DNAH9* mutations and altered cytokine profiles that may support lung lesion development. Previous research revealed that higher levels of Matrix metalloproteinases (MMPs) and lower concentrations of TIMPs were associated with airway remodeling at least in half of the children and in two third of the adults with PCD [24]. Mycoplasma pneumoniae in adult community-acquired pneumonia increases matrix metalloproteinase-9 serum level and induces its gene expression in peripheral blood mononuclear cells [25, 26]. Although children with *DNAH9* mutations typically exhibit milder respiratory symptoms, it is may because that DNAH9 is located at the distal end of the cilia, where the cilia still retain partial motile function. In the absence of infection, they can maintain adequate airway clearance function. However, during virus and/or Mycoplasma pneumoniae (MP) infections, excessive airway inflammation and epithelial damage are triggered. This may achieve by inducing the release of inflammatory factors, modulating goblet cell mucus secretion in the airways, and increasing the level of matrix metalloproteinases, thereby leading to airway remodeling and epithelial-mesenchymal transition regeneration which finally lead to BO.

In summary, findings from this and other studies emphasize the pivotal role of DNAH9 variants in PIBO pathogenesis. However, further recruitment of PIBO cases with *DNAH9* variants is needed to broaden our comprehension of PCD phenotypes. Additionally, more investigations are warranted to elucidate PIBO pathogenic mechanisms, explore genotype–phenotype correlations, understand the interplay between PIBO and ciliary dysfunction, and unravel the pathophysiological mechanisms associated with DNAH9 dysfunction.

## Data Availability

The datasets analyzed for this study are available from the first author Dr.Yuhong Guan (guanyuhong123@126.com) upon reasonable request.

## References

[1] Morris-Rosendahl DJ. Primary ciliary dyskinesia as a common cause of bronchiectasis in the Canadian Inuit population. Pediatr Pulmonol. 2023;58(9):2437–8. 37278553 10.1002/ppul.26529

[2] Mitchison HM, Smedley D. Primary ciliary dyskinesia: a big data genomics approach. Lancet Respir Med. 2022;10(5):423–5. 35051410 10.1016/S2213-2600(22)00009-1

[3] Loges NT, Antony D, Maver A, Deardorff MA, Güleç EY, Gezdirici A, Nöthe-Menchen T, Höben IM, Jelten L, Frank D, Werner C, Tebbe J, Wu K, Goldmuntz E, Čuturilo G, Krock B, Ritter A, Hjeij R, Bakey Z, Pennekamp P, Dworniczak B, Brunner H, Peterlin B, Tanidir C, Olbrich H, Omran H, Schmidts M. Recessive DNAH9 Loss-of-Function mutations cause laterality defects and subtle respiratory Ciliary-Beating defects. Am J Hum Genet. 2018;103(6):995–1008. 30471718 10.1016/j.ajhg.2018.10.020 PMC6288205

[4] Fassad MR, Shoemark A, Legendre M, Hirst RA, Koll F, le Borgne P, Louis B, Daudvohra F, Patel MP, Thomas L, Dixon M, Burgoyne T, Hayes J, Nicholson AG, Cullup T, Jenkins L, Carr SB, Aurora P, Lemullois M, Aubusson-Fleury A, Papon JF, O’Callaghan C, Amselem S, Hogg C, Escudier E, Tassin AM, Mitchison HM. Mutations in outer dynein arm heavy chain DNAH9 cause motile cilia defects and situs inversus. Am J Hum Genet. 2018;103(6):984–94. 30471717 10.1016/j.ajhg.2018.10.016 PMC6288320

[5] Tang D, Sha Y, Gao Y, Zhang J, Cheng H, Zhang J, Ni X, Wang C, Xu C, Geng H, He X, Cao Y. Novel variants in DNAH9 lead to nonsyndromic severe asthenozoospermia. Reprod Biol Endocrinol. 2021;19(1):27. 33610189 10.1186/s12958-021-00709-0 PMC7896388

[6] Dizier MH, Nadif R, Margaritte-Jeannin P, Barton SJ, Sarnowski C, Gagné-Ouellet V, Brossard M, Lavielle N, Just J, Lathrop M, Holloway JW, Laprise C, Bouzigon E, Demenais F. Interaction between the DNAH9 gene and early smoke exposure in bronchial hyperresponsiveness. Eur Respir J. 2016;47(4):1072–81. 26797031 10.1183/13993003.00849-2015

[7] Flanagan F, Casey A, Reyes-Múgica M, Kurland G. Post-infectious bronchiolitis obliterans in children. Paediatr Respir Rev. 2022;42:69–78. 35562287 10.1016/j.prrv.2022.01.007

[8] Duecker RP, De Mir Messa I, Jerkic SP, Kochems A, Gottwald G, Moreno-Galdó A, Rosewich M, Gronau L, Zielen S, Geburtig-Chiocchetti A, Kreyenberg H, Schubert R. Epigenetic regulation of inflammation by MicroRNAs in post-infectious bronchiolitis obliterans. Clin Transl Immunol. 2022;11(2):e1376. 10.1002/cti2.1376 PMC8859819 35228871

[9] Şişmanlar Eyüboğlu T, Aslan AT, Ramaslı Gürsoy T, Pekcan S, Köse M, Hangül M, Aral LA, Bulut V. Caspase-1 and interleukin-18 in children with post infectious bronchiolitis obliterans: a case-control study. Eur J Pediatr. 2022;181(8):3093–101. 35705877 10.1007/s00431-022-04528-2

[10] Giubergia V, Salim M, Fraga J, Castiglioni N, Sen L, Castaños C, Mangano A. Post-infectious bronchiolitis obliterans and mannose-binding lectin insufficiency in Argentinean children. Respirology. 2015;20(6):982–6. 25939617 10.1111/resp.12547

[11] Guan Y, Yang H, Yao X, Xu H, Liu H, Tang X, Hao C, Zhang X, Zhao S, Ge W, Ni X. Clinical and genetic spectrum of children with primary ciliary dyskinesia in China. Chest. 2021;159(5):1768–81. 33577779 10.1016/j.chest.2021.02.006 PMC8129725

[12] Caballero-Colón NM, Guan Y, Yang H, Zhao S, De Jesús-Rojas W. Bronchiolitis obliterans and primary ciliary dyskinesia: what is the link?? Cureus. 2021;13(6):e15591. 34277212 10.7759/cureus.15591 PMC8272919

[13] Kavaliunaite E, Aurora P. Diagnosing and managing bronchiolitis obliterans in children. Expert Rev Respir Med. 2019;13(5):481–8. 30798629 10.1080/17476348.2019.1586537

[14] Colom AJ, Teper AM. Post-infectious bronchiolitis obliterans. Pediatr Pulmonol. 2019;54:212–9. 30548423 10.1002/ppul.24221

[15] Zheng R, Yang W, Wen Y, Xie L, Shi F, Lu D, Luo J, Li Y, Zhang R, Chen T, Chen L, Xu W, Liu H. Dnah9 mutant mice and organoid models recapitulate the clinical features of patients with PCD and provide an excellent platform for drug screening. Cell Death Dis. 2022;13(6):559. 35729109 10.1038/s41419-022-05010-5 PMC9210797

[16] Chen W, Zhang Y, Shen L, Zhu J, Cai K, Lu Z, Zeng W, Zhao J, Zhou X. Biallelic DNAH9 mutations are identified in Chinese patients with defective left-right patterning and cilia-related complex congenital heart disease. Hum Genet. 2022;141(8):1339–53. 35050399 10.1007/s00439-021-02426-5

[17] Takeuchi K, Xu Y, Ogawa S, Ikejiri M, Nakatani K, Gotoh S, Usui S, Masuda S, Nagao M, Fujisawa T. A pediatric case of productive cough caused by novel variants in DNAH9. Hum Genome Var. 2021;8(1):3. 33452233 10.1038/s41439-020-00134-6 PMC7810879

[18] Isa HM, Alkharsi FA, Busehail MY, Haider F. A novel DNAH9 gene mutation causing primary ciliary dyskinesia with an unusual association of jejunal Atresia in a Bahraini child. Cureus. 2022;14(12):e32964. 36712782 10.7759/cureus.32964 PMC9876387

[19] Flanagan F, Casey A, Reyes-Mugica M, Kurland G. Post-infectious bronchiolitis obliterans in children. Paediatr Respir Rev. 2022;42:69–78. 35562287 10.1016/j.prrv.2022.01.007

[20] Hildebrandt GC, Granell M, Urbano-Ispizua A, Wolff D, Hertenstein B, Greinix HT, Brenmoehl J, Schulz C, Dickinson AM, Hahn J, Rogler G, Andreesen R, Holler E. Recipient NOD2/CARD15 variants: a novel independent risk factor for the development of bronchiolitis obliterans after allogeneic stem cell transplantation. Biol Blood Marrow Transpl. 2008;14(1):67–74. 10.1016/j.bbmt.2007.09.009 18158963

[21] Huang F, Ma YC, Wang F, Li YN. Clinical analysis of adenovirus postinfectious bronchiolitis obliterans and nonadenovirus postinfectious bronchiolitis obliterans in children. Lung India. 2021 Mar-Apr;38(2):117–121. 10.4103/lungindia.lungindia_374_20 PMC8098888 33687003

[22] Tamiya S, Yoshikawa E, Ogura M, Kuroda E, Suzuki K, Yoshioka Y. Neutrophil-Mediated lung injury both via TLR2-Dependent production of IL-1α and IL-12 p40, and TLR2-Independent CARDS toxin after Mycoplasma pneumoniae infection in mice. Microbiol Spectr. 2021;9(3):e0158821. 34937175 10.1128/spectrum.01588-21 PMC8694186

[23] Lee E, Park S, Kim K, Yang HJ. Risk factors for the development of Post-Infectious bronchiolitis obliterans in children: A systematic review and Meta-Analysis. Pathogens. 2022;11(11):1268. 36365019 10.3390/pathogens11111268 PMC9696236

[24] Pifferi M, Bush A, Caramella D, Metelli MR, Di Cicco M, Piras M, Gherarducci G, Capristo C, Maggi F, Peroni D, Boner AL. Matrix metalloproteinases and airway remodeling and function in primary ciliary dyskinesia. Respir Med. 2017;124:49–56. 28284321 10.1016/j.rmed.2017.02.001

[25] Caughey GH. Matrix metalloproteinase-2 and– 9 expression increases in Mycoplasma-infected airways but is not required for microvascular remodeling. Am J Physiol Lung Cell Mol Physiol. 2004;287(2):L307–17. 15075248 10.1152/ajplung.00404.2003

[26] Puljiz I, Markotić A, Cvetko Krajinovic L, Gužvinec M, Polašek O, Kuzman I. Mycoplasma pneumoniae in adult community-acquired pneumonia increases matrix metalloproteinase-9 serum level and induces its gene expression in peripheral blood mononuclear cells. Med Sci Monit. 2012;18(8):CR500–505. 22847199 10.12659/MSM.883270 PMC3560704

